# Book Reviews

**Published:** 1806-05

**Authors:** 


### CRITICAL ANALYSIS
### OF THE
### RECENT PUBLICATIONS
### ON THE
### DIFFERENT BRANCHES OF PHYSIC, SURGERY, AND MEDICAL PHILOSOPHY.

---

*A Treatiſe on the Origin, Progreſs, Prevention, and Treatment of Conſumption.* By JOHN REID, M. D. Member of the Royal College of Phyſicians, London, Senior Phyſician to the Finſbury Diſpenſary, and Lecturer on the Theory and Practice of Medicine. 8vo. London.

IN the Preface to this Work we meet with the following ſtatement of the views with which it was compoſed. " The writer of the enſuing pages has laboured to render the diſquiſitions that they contain, at the ſame time open to general obſervation, and intereſting to the profeſſional ſtudent. He has attempted to delineate a portrait, the prominent character of which ſhall be univerſally underſtood, while the more minute lines and ſhades of expreſſion ſhall remain for detection by the ſkill of the artiſt."

In purſuance of this plan, our author introduces his ſubject with a ſlight ſketch of thoſe different hypotheſes which have, at various periods, been conſtructed in order to explain the nature and origin of what he conceives neceſſary to the actual exiſtence of genuine phthiſis, ulcer in the lungs, and hectic fever. The principal errors of ſuch hypotheſes are attributed, by Dr. Reid, to a neglect on the part of their founders, of regarding life as an effect, produced and ſupported by influences totally diſtinct in their nature from thoſe which regulate the economy of the inanimate creation.

" Theories of diſeaſe were deduced from the principles of looſe analogical analogical reafoning; unprofitable difquifitions into the caufe, for a long period, fupplied the place of an attentive inquiry into the laws and characteriftic qualities of vitality, and the fcience of life remained to be difclofed."

That reformation, fo long defired in the conduct of medical refearches, has at length been effected, according to the opinion of our author, by the enunciation and application of what is generally denominated the Brunonian theory, the outlines of which are defcribed by the following words: "Every change which is effected on the folids or fluids of the living fyftem, is fubordinate to, and confequent upon, a certain degree of action in the animate fibre. This action is produced upon principles entirely different from the impulfe of mechanifm, or the attractions of inanimate matter. It is governed by peculiar and exclufive laws. The powers by which it is generated are the powers by which life is fuftained. Health confifts in its regular maintenance. The various modifications of difeafe are in their origin entirely attributable to its derangement, and death is the inevitable and immediate confequence of the lofs of fufceptibility to its action."

The four fucceeding chapters are devoted to a delineation of the ftructure of the refpiratory organs, an account of the chemical ingredients of atmofpheric air, and a general inquiry into the functions of the lungs and fkin, as far as they are connected with the propagation and prefervation of animal heat. Our limits will not permit us to fcrutinize the reafonings of Dr. Reid, from which the following inference is deduced, that chemical agency has been too precipitately made to apply to the functions both of the lungs and external furface of the body; and that the generation and due adjuftment of animal temperature, are, like all other phenomena of the living frame, influenced by the peculiar laws of fibrous excitation.

Thofe affections are next made to pafs under review, which are frequently precurfors of ulcer in the lungs; thefe are treated of in the following order, *Hæmoptyfis*, *Catarrh*, *Pneumonia*, and *Tubercles*.

In the chapter on Hæmoptyfis, Dr. R. takes ocafion to object to thofe accounts of its origin, which he conceives to be relicks of the mechanical and humoral doctrines in pathology. Difcharges of blood from the lungs, our author maintains, in no inftance originate according to the opinion of Dr. Cullen and other writers, from a fudden diminution of weight in the atmofphere, acting either upon the principles of mechanifm, or by occafioning the circulating fluids to rarify and expand; an effect which he contends is altogether inconfiftent with the eftablifhed laws of vital caufation. While confidering the treatment of hæmorrhage from the lungs, as likewife the production and treatment of catarrh and pneumonia, the writer's principles are maintained with much ingenuity of argument, and confiderable force of expreffion.

We were much gratified with obferving, that in his remarks on pneumonia, our author adverts, in an efpecial manner, to the tendency of eruptive diforders in early life, more efpecially the mea-

ſles, to aſſiſt or even create phthiſical diſpoſition, unleſs immediately and effectually counteracted; for the purpoſe of preventing this occurrence, he is of opinion, with many contemporary writers, that Digitalis furniſhes an important agent.

With reſpect to tubercles, Dr. R. objects to that vague mode of explanation, which refers their origin to conſtitutional acrimony; but does not, in our opinion, ſubſtitute any theory of their production which is explicit or ſatisfactory. It is not ſufficient to deſcribe theſe bodies, as conſequent upon ſlight and repeated inflammation, depoſiting matter which remains unabſorbed. We conceive the queſtion at iſſue to be, Why ſhould that peculiar depoſition, by which a tubercle is conſtituted, reſult from inflammation in parenchymatous ſubſtance excluſively? To aſcribe this to "peculiarity of ſtructure," is not to explain, but merely to ſtate its cauſe.

We are, moreover, diſpoſed to think that Dr. R. has evinced too great a partiality for ſyſtem and generalization, in his endeavours to prove that ſuſceptibility to catarrhal irritation of the mucous membrane, and that the generation of tubercle, exiſt in the ſame ratio. Violent and reiterated inflammation, we are perſuaded, frequently affect the mucous membrane without being followed by tubercle.

In the next diviſion of his work, Dr. R. conſiders the prediſpoſing and exciting cauſe of conſumption; and the characters of the phthiſical temperament are here pointed out with ſo much perſpicuity and accuracy, that we regret our limits do not admit of their detail.

On the means of obviating the conſumptive tendency, we find a conſiderable coincidence of opinion with another modern author of deſerved reputation; a coincidence which will in no meaſure detract from the value of thoſe precepts which are contained in this important diviſion of Dr. R's work.

The progreſſive ſymptoms of phthiſis pulmonalis are traced with fidelity in the enſuing chapter, and the practical rules, with regard to the treatment of the complaint, diſplay much nicety of diſcrimination, and correctneſs of judgment. We conceive, however, that the author would have rendered his treatiſe more acceptable to the majority of readers, by a more ample detail of both the dietetic and medicinal management of the conſumptive invalid. In the earlier ſtages of this complaint, Dr. R. ſpeaks with much confidence of the powers of Digitalis, the modus operandi of which he endeavours to reconcile with Brunonian principles.

The work now under conſideration concludes with additional obſervations on the mode of diſcriminating between genuine and ſpurious phthiſis, with an account of the different ſpecies and cauſes of general decline, and pulmonary conſumption; and with further remarks on the remedies required in conſumptive affections, whether independent of, or connected with, an actual diſorder of the lungs.

We ſhall finiſh our Analyſis by extracting the following paragraphs, graphs, which terminate the work, and which we think afford a favourable ſpecimen of the general ſtyle in which it is written.

"Coughs are by no means abſolute indications of catarrhal affection. The idea of 'ſpecific remedies for coughs and colds,' is founded upon a total ignorance of the laws and functions of the animal economy. It is equally inconſiſtent with, and contrary to, both philoſophical principles and actual experience, as the vulgar and empirical notion of correcting impurities in the blood.

"Urgent pain is often unaccompanied by inflammation or increaſe of local excitement. It is in many inſtances conſequent upon the difficulty and labour with which an enfeebled organ performs its accuſtomed and ſalutary action. An important demand for invigorating reſources is too commonly ſupplied by enervating powers.

The fluſhed cheek and emaciated countenance, are the moſt faithful attendants upon genuine phthiſis. Even theſe, however, are not abſolute indications either of the preſence or abſence of pulmonary conſumption.

"A familiarity with the phyſiognomy of this diſeaſe can only be acquired by an aſſiduous attention to its ever varying ſhades, and by carefully marking its multifarious expreſſions. In proportion, however, to the difficulty of the taſk is the neceſſity of its accompliſhment. Delays and errors are here eſpecially to be dreaded. Deciſion comes too late, when not merely the nature of the diſorder, but likewiſe its fatal termination, almoſt ceaſes to be a ſubject of doubt or enquiry. To know the rocks on which his veſſel has foundered, affords but ſmall conſolation to the ſhipwrecked mariner."

---

*A Treatise on the Process employed by Nature in suppressing the Hemorrhage from divided Arteries, and on the Use of the Ligature; concluding with Observations on secondary Hemorrhage; the whole deduced from an extensive Series of Experiments, and illusrated by fifteen Plates.* By J. F. D. Jones, M. D. Member of the Royal College of Surgeons of London.

We know not whether most to admire the benevolent intentions of this author, his persevering industry, or the accuracy and minuteness of his observation under so long a series of experiments. If ever there was a subject which authorized experiments on brute animals more than another, it is the one before us. It was not with a view of establishing some uncertain theory, some unimportant doctrine, the favourite invention of his brain, but from the sole motive of relieving his fellow creatures under circumstances which admit of no delay, that these enquiries and communications have been made.

That our readers may be enabled to follow us through the rest of the work, we shall transcribe the first chapter, entitled, *preparatory considerations.*

"The changes produced on arteries by accidents, and the surgical gical operations to which they are subjected, have a certain relation to their structure. The subjects, therefore, of this Treatise naturally suggest a few preliminary observations on the structure of arteries; but only such a description will be given, as may suffice for the explanation of the phænomena which they exhibit, when influenced by accident or art.

"The substance of which arteries are composed is divisible into distinct parts, which have been called tunics or coats. Three coats, which have received various names, can be readily demonstrated, and may be simply and clearly distinguished by the terms —Internal, Middle, and External.

"The internal coat, although extremely thin, is very close in its texture, and gives to an artery a smooth and polished lining; it is elastic, and firm, considering its delicate structure, in the longitudinal direction, *but so weak in the circular as to be very easily torn by the slightest force applied in that direction.* The morbid changes, which have been observed in it, prove that this coat is vascular; and some experiments have been related to show the probability of its being sensible.

"The middle coat, which is the thickest, is formed by numerous layers of firm, compact, fleshy fibres of a pale red colour, passing in a circular direction, but appearing rather obliquely connected and interlaced with each other, than forming complete circles. These fibres are of a peculiar nature, are well supplied with nerves, and resemble in form and disposition, muscular fibres, but differ from them in possessing a remarkable degree of elasticity. Their elasticity keeps an empty and dead artery open and circular; for this coat, when detached from the internal and external coats, still preserves a cylindrical form, whilst they, on the contrary, in a state of separation, become flaccid and collapse. As this coat has no longitudinal fibres, *the circular fibres are held together by a slender connection, which yields readily to any force applied in the circumference of the artery.* The middle coat is intimately connected with the internal and external by very short and fine cellular membrane.

"The external coat, anatomically considered, is so simple, that many authors have thought it sufficient to say, that it is formed of condensed cellular membrane, which becoming gradually of a looser texture, connects the artery with the surrounding parts; but the importance which is attached, in a surgical view, to this coat, renders a more particular account of it highly necessary and interesting. Although ultimately resolvable into cellular membrane, yet it derives from the particular arrangement of its component fibres a characteristic appearance, which distinguishes it from cellular membrane, and entitles it to be ranked as a proper coat of an artery. Internally, or next to the middle coat, its texture is close and smooth, externally more open and rough, in consequence of the cellular membrane by which it is connected with an additional covering. The whole is remarkable for its whiteness, ness, density, and great elasticity. If an artery be surrounded by a tight ligature, its middle and internal coats will be as completely divided by it as they can be by a knife, whilst the external coat remains entire; a fact, which will be commented upon in another part of this treatise, and shewn to be connected with important circumstances. The strength therefore of an artery depends chiefly on it s external coat, which answers, in some respects, the purpose of a strong fascia.

"The three coats which I have just described are the only proper coats of an artery; but the different arteries, while in their natural situation, are surrounded with fine cellular membrane that connects them with additional coverings, called sheaths, which are formed within the cavities of the body by the investing membrane peculiar to each, and in other parts by facia, or cellular membrane. The fine cellular membrane, the bond of connection between arteries and their sheaths, varies in length in different parts, and gives an artery a flocculent appearance. If an artery be divided, the divided parts, owing to their elasticity, recede from each other, and the length of the cellular fibres connecting the artery with the sheath, admits of its retracting a certain way within the sheath; an important fact, the application of which will be made in a future part of this Treatise, that describes the natural process by which hemorrhage is stopped.

"In performing the experiments, which will be subsequently related, it was not my intention to investigate the long agitated question respecting the action of arteries, whether it should be ascribed to their elasticity alone, or their elasticity and muscularity conjointly. I have had, however, frequent opportunities, in the course of my experiments, of observing appearances, which incline me strongly to the latter opinion, slightly modified. These appearances made so conspicuous a part of the process, to which my attention was more immediately directed, that I was induced to discuss the subject at some length in my Thesis. Here we are only concerned to notice one important fact relating to the action of arteries, namely: that when an artery is divided, its truncated extremities contract in a greater or less degree, and the contraction is generally if not always permanent. But I have only once seen a distinct contraction, produced by a mechanical stimulus, while the artery remained entire. It happened in an artery on which the effect of galvanism was tried, but it was uncertain, even in this instance, whether the contraction should be referred to the irritation of the wire of the galvanic pile, or to the galvanic influence, which it conveyed.

Arteries are supplied not only with small arteries and veins (the vasa vasorum), but also with absorbents and nerves, and have, in these respects, a similar organization to the other soft parts of the body. This structure makes them susceptible of every change to which living parts are subjected in common; enables them to inflame inflame when injured, and to pour out coagulating lymph, by which the injury is repaired, or the tube is permanently closed."

This account, when we consider that the whole of the future experiments are on arteries, is much too short. We admit the caution of our author in not asserting more than he could prove. He observes, with much propriety, that the elasticity of these fibres, which run in a somewhat circular direction, is the means of preserving the cavity of a dead artery: but as he found that the artery had a considerable degree of elasticity, in its longitudinal direction, so as to shorten itself when cut, it became him to define the degree and nature of these elastic powers. By determining how much power remained in a dead artery, and comparing it with the living artery, he might ascertain this as far as the subject would admit; the power lost after death being subtracted from what remained, would show the degree of muscular power, which could only exist with life. What remained must be considered as elastic. It was not necessary, however, to pursue this subject experimentally to any extent. Mr. Hunter's experiments are so numerous, and his accuracy so well known, that a few, to strengthen or confute them, would have been sufficient. The only objection we have to the passage above quoted is, that the author assumes these muscular and elastic powers of arteries, without offering any proofs either from himself, or any other writer. In all other respects, this chapter is highly important, and must be constantly kept in view by the reader, particularly the description of the cellular sheath, and the manner in which a divided artery contracts within it.

The first section is on the process employed by Nature, for suppressing hemorrhage from divided arteries, or, as we should prefer expressing it, on the sources of the constitution, or animal economy, for suppressing, &c.

In the first chapter the author gives a very candid and accurate review of the Theories offered by Petit, Morand, Sharp, Porteau, Gooch, Kirkland, White, and J. Bell. In this we were much pleased to observe the just respect shown to those old French writers, to whom we owe so much; but our obligations to whom are so seldom acknowledged. Mr. Petit, it is observed, perceiving a coagulum lodged at the inside of the extremity of a divided artery, and also that the external part was immersed in a similar substance, concluded that the plug within, and the support without, were the causes of suppressed hemorrhage. M. Morand added to the above causes, the contraction and retraction of divided arteries. The opinions of both these gentlemen, our author, with a becoming candour, shows, were founded on fact, though as he adds, their opportunities did not allow them to pursue their enquiries far enough. Mr. Sharpe's opinion, as expressed in the Edition of 1783, is nearly similar to the above.

Dr. Jones begins his remarks on M. Porteau's account, by regretting how much the value of it is lessened by the severity with which which his predecessors are treated. M. Porteau, by making similar experiments to those of Petit, found similar results; but in stumps which he had an opportunity of examining at different distances of time, after amputation, he perceived that in some, no coagulum was discoverable within the cavity of the artery, and in others, where coagula appeared, neither its firmness nor adhesion to the vessel, were sufficient to prevent hemorrhage. This induced him to impute the whole to the contraction of the length and corrugation or constriction of the diameter of the artery.

Mr. Gooch's readiness at adopting Porteau's error, as his own discovery, is next delicately touched upon, and also Mr. Kirkland's opinion, that the suppression of hemorrhage was caused solely by the contraction of the arterial diameter; in which he was implicitly followed by Mr. White and Mr. Aikin. But though Mr. White's principal, if not his only concern, should have been with the unassisted powers of the economy, his conclusions are drawn from the condition of a tied artery.

Mr. J. Bell's Theory, being the last that has been offered, and the most likely to interest the rising generation; we shall offer our author's remarks upon it, in his own words.

"The last theory which I have to notice, is that lately published by Mr. J. Bell, who, after freely criticising those which I have already mentioned, confidently asserts, that, "when hemorrhage stops of its own accord, it is neither from the retraction of an artery, nor the constriction of its fibres, nor the formation of clots, but by the cellular substance which surrounds the artery being injected with blood." It is to be regretted, that among the plates which Mr. Bell has very judiciously introduced to illustrate the doctrines of Petit and Pouteau, he has not added one to exemplify his own, which, although it appears to be delivered in very decisive terms, yet, in its affirmative part at least, is vague and inconclusive. It was perhaps on this account that the author subjoined a copious illustration of it, which I shall also quote, that I may not incur the reproach of having given only a partial and unjust view of his theory.

"The stream of blood gradually lessens, because the artery is emptied, and the resistance to the arterial action taken away; the stimulus being gradually lessened, the artery every moment acts less powerfully; and the blood being no longer solicited or urged on by the arterial contractions, forsakes the open artery, and moves along the neighbouring branches. The surgeon claps the point of his finger upon the mouth of the artery, and holds it there; the outward bleeding is prevented; the blood is extravasated into the cellular substance round the mouth of the artery; the cellular substance is slightly injected with blood; that blood coagulates, and that slight barrier is sufficient to restrain the bleeding of a small artery, till the parts inflame, and the artery is entirely stopped.

"Supposing

"Supposing the artery still larger and more powerful, and "that it drives its blood very furiously among the cellular sub- "stance, it is not this slight injection of the cellular substance "that will restrain the bleeding. Whenever the finger is re- "moved the blood bursts through this slight impediment. The "injected cellular substance will not support the artery, unless "the cellular substance itself be also supported. . . . .

" . . . . . . . . . . Retraction of the artery has no effect in "suppressing hemorrhagy, but as it fills the cellular substance; and this injection of the cellular substance is but a slight obstacle, "fit to support only the very smallest arteries. The natural "powers which restrain hemorrhagy, do but suppress it for a "time, and expose the patient to secondary hemorrhage."

"According to Mr. Bell's first position it appears, that the injection of blood into the cellular membrane which surrounds the artery, is the only natural cause, by which hemorrhage is stopped; but as he has just before said that it is not stopped by the formation of clots, we are at a loss to know how this injection of the cellular membrane effects the suppression of hemorrhage. If we have recourse to his explanation, our curiosity is not gratified, and our embarrassment certainly not diminished, for we there find that the cellular membrane is only "slightly injected with "blood," so that we are not at liberty to infer that it is so completely injected as to compress the artery, and in that way stop the flow of blood. Indeed, however plausible such an assertion might have made the doctrine, it would not have been valid: but, continues the author, "that blood coagulates, and "that slight barrier is sufficient to restrain the bleeding of a small "artery, &c." But what is the nature of this barrier? and in what manner does it stop the hemorrhage? for it is to be remembered that we are previously informed, in positive terms, that it is not stopped by the formation of clots; and although, in conformity with this, it is not said that the blood is effused into the cellular membrane, *at the mouth* of the artery, but round it, yet from what has been said above, it must, I think, be pretty evident that an effectual compression of the artery is not implied.

"Left then, as we have been by the author, to make the best we can of the theory and explanation, the only conclusion at which we can arrive without assistance from him, is, that hemorrhage is not stopped "by the formation of clots, but by the cellular substance, which surrounds the artery, being injected with blood, which, according to the illustration, coagulates;" but as the artery is every where surrounded with cellular substance, the coagulated blood, which stops the hemorrhage, must necessarily be in the cellular substance; we have, therefore, only to discuss the difference between a clot of blood and coagulated blood, to discover the principal difference between this offspring of Mr. Bell, and what he has been pleased to call Petit's "sickly child."

"Retraction of the artery," says Mr. Bell, "has no effect in suppressing suppressing hemorrhagy, but as it fills the cellular substance." But let it be observed, that besides filling the cellular substance round the artery, it also fills the cellular substance at the mouth of the artery in a particular manner; for the divided artery, by its retraction within its cellular sheath, leaves a space of a determinate form, which, all the circumstances necessary for the suppression of hemorrhage operating, is gradually filled up by a distinct clot. If Mr. Bell really means to confine his doctrine of the natural means of suppressing hemorrhage to the injection of the cellular membrane round the artery with blood; he dwells improperly on one of the attendant circumstances to the exclusion of the retraction and contraction of an artery, and the formation of a distinct clot—all of them primary means in the natural suppression of hemorrhage, of which abundant proofs will be given in the proper place.

"I have chosen to set Mr. Bell's theory in its strongest and best point of view, by confining it to the injection of the cellular membrane with blood; for, in justice to his physiological knowledge, I think it unnecessary to examine the accuracy of the reasoning, by which he would shew, that the blood *forsakes* the *open* artery; rather supposing, that, on this occasion, he has hastily adopted the account given by the Editor of the Memoires de l'Academie Royale des Sciences for the year 1735; who, in giving an abstract of Petit's paper, for the year 1731, says, "Dans le cas d'un tronc d'artere coupé, *le sang qui continue de s'y rendre, ne doit plus y couler que jusqu'a l'endroit, ou il rencontrera une branche collaterale entiere dont il enfilera la route,* au moyen de quoi la circulation s'achevera." And I am still less disposed to dwell on the inconsistency of introducing the surgeon's finger as an auxillary to the *natural* means by which hemorrhage is stopped."

After this account of the labours of his predecessors, our author begins the history of his "experiments on the arteries of dogs and horses, to ascertain the process, and the order of the events which constitute it." We shall not enter into a detail of these at present, reserving our remarks on some particular passages to the conclusion drawn from the experiments. Suffice it to say, that equal industry and judgment are shown in every part; that the result is perfectly satisfactory; that if there is any thing to which we object, it is, adopting Dr. Jones's own expression, "to the manner of accounting for the appearances which he observed." However, as we cannot add, that "his doctrine could only have been deduced from the irregular and partial observations which he was enabled to make," we would not wish that our objections should be considered in any other light than as opinions, which the reader will receive or reject as he pleases.

"The results (says our author) of the experiments related in the last section will not allow us to give so conscise and simple an account of the process, as has hitherto been done; but they afford us us one more satisfactory, becaue it accords better with the operations of the animal œconomy, in which we are accustomed to observe the most important changes gradually produced by the co-operation of several means, rather than by the sole influence of any one in particular.

"They accordingly shew, that the blood, the action and even the structure of arteries, their sheath, and the cellular substance connecting them with it; in short, that all the parts concerned in or affected by hemorrhage, contribute to arrest its fatal progress, by operating, in the case of a divided artery of moderate size, in the following manner.

"An impetuous flow of blood, a sudden and forcible retraction of the artery within its sheath, and a slight contraction of its extremity, are the immediate and almost simultaneous effects of its division. The natural impulse, however, with which the blood is driven on, in some measure counteracts the retraction, and resists the contraction of the artery. The blood is effused into the cellular substance between the artery and its sheath, and passing through that canal of the sheath which had been formed by the retraction of the artery, flows freely externally, or is extravasated into the surrounding cellular membrane, in proportion to the open or confined state of the external wound. The retracting artery leaves the internal surface of the sheath uneven by lacerating or stretching the cellular fibres that connected them. These fibres entangle the blood as it flows, and thus the foundation is laid for the formation of a coagulum at the mouth of the artery, and which appears to be completed by the blood, as it passes through this canal of the sheath, gradually adhering and coagulating around its internal surface, till it completely fills it up from the circumference to the centre.

"A certain degree of obstruction to the hemorrhage, which results from the effusion of blood into the surrounding cellular membrane, and between the artery and its sheath, but particularly the diminished force and velocity of the circulation, occasioned by the hemorrhage, and the speedy coagulation of the blood, which is a well known consequence of such diminished action of the vascular system, most essentially contribute to the accomplishment of this important and desirable effect.

"A coagulum then, formed at the mouth of the artery, and within its sheath, and which I have distinguished in the experiments by the name of the external coagulum, presents the first complete barrier to the effusion of blood. This coagulum, viewed externally, appears like a continuation of the artery, but on cutting open the artery, its termination can be distinctly seen with the coagulum completely shutting up its mouth, and inclosed in its sheath.

"The mouth of the artery being no longer pervious, nor a collateral branch very near it, the blood just within it is at rest, coagulates, and forms, in general, a slender conical coagulum, which which neither fills up the canal of the artery, nor adheres to its sides, except by a small portion of the circumference of its base, which lies near the extremity of the vessel. This coagulum is distinct from the former, and I have called it the internal coagulum.

" *In the mean time the cut extremity of the artery inflames, and the vasa vasorum pours out lymph, which is prevented from escaping by the external coagulum. This lymph fills up the extremity of the artery, is situated between the internal and external coagula of blood, is somewhat intermingled with them, or adheres to them, and is firmly united all round to the internal coat of the artery.*

" The permanent suppression of the hemorrhage chiefly depends on this coagulum of lymph; but while it is forming within, the extremity of the artery is farther secured by a gradual contraction which it undergoes, and by an effusion of lymph between its tunics, and into the cellular membrane surrounding it; in consequence of which these parts become thickened, and so completely incorporated with each other, that it is impossible to distinguish one from the other: thus, not only is the canal of the artery obliterated, but its extremity also is completely effaced, and blended with the surrounding parts.

" When the wound in the integuments is not healed by the first intention, coagulating lymph, which is soon effused, not only attaches the artery firmly to the subjacent and lateral parts, but also gives it a new covering, and completely excludes it from the external wound, which then goes on to fill up and heal in the usual manner.

" The circumstances now described are observed also in the inferior portion of the artery, or that which is supplied with blood by anastomosis; with this difference only, that its orifice is generally more contracted, and the external coagulum is much smaller than the one which adheres to the mouth of the superior portion of the artery, or that from which the blood flows in its direct course from the heart.

" From this view of the subject we can no longer consider the suppression of hemorrhage as a simple or mere mechanical effect, but as a process performed by the concurrent and sucessive operations of many causes: these may briefly be stated to consist in the retraction and contraction of the artery; the formation of a coagulum at its mouth; the inflammation and consolidation of its extremity by an effusion of coagulating lymph within its canal, between its tunics and in the cellular substance surrounding it.

" And we may conclude that, except in some rare instances, in which the strong retraction and contraction of a divided or lacerated artery prevents hemorrhage altogether, a languid state of the circulation is necessary for the accomplishment of the natural means by which the hemorrhage is stopped. These means may be divided into the *temporary* and *permanent*: under the former head we may include the three first of the above-mentioned causes; causes; whilst the effusion of lymph constitutes the permanent: yet even these can be distinctly traced only for a certain time, in consequence of other changes which the artery gradually undergoes. Its obliterated extremity no longer allowing the blood to circulate through it, the portion which lies between it and the first lateral branch is no more distended and excited to action as formerly; but gradually contracts, till at length its cavity is completely obliterated, and its condensed tunics assume a ligamentous appearance. At the same time, the remarkable appearances at the extremity of the artery are undergoing a considerable change; the external coagulum of blood, which in the first instance had stopped the hemorrhage, is absorbed in the course of a few days, and the coagulating lymph, which had been effused around it, and had produced a thickened and almost cartilaginous appearance in the parts, is gradually removed, and they again appear more or less completely restored to their cellular texture.

"Nor are these all the changes which the artery undergoes; for, if examined at a still later period, the ligamentous portion is found to be reduced to a filamentous state, distinguishable from the surrounding cellular membrane only by being somewhat coarser; and thus the obstruction which commenced at the extremity of the canal terminates in the complete annihilation of the artery to the first lateral branch.

"But long before this final change is accomplished, many of the lateral branches of the superior and inferior portions of the artery have become very much enlarged, and have established by frequent anastomoses, a free and ready communication between these disunited parts of the trunk. The small branches by whose immediate inosculation these anastomoses are formed, appear to have undergone the principal changes; they are not only proportionably more enlarged than the large branches of the limb to which they belong, and very considerably larger than the corresponding branches of the other limb, but have also become longer, and, being confined within their former space, assume a beautiful tortuous and serpentine course, in order to accommodate themselves to it.

"The circulation appears to be carried on as perfectly and vigorously by these anastomising branches in the limb, the main artery of which has been divided, as in that in which the artery is entire; the inferior part of the divided artery, and all its branches, being found fully equal in size to the corresponding part of the trunk and branches of the artery of the opposite limb which has not been divided: and hence, we may conclude, with the celebrated Mr. Hunter, that "Vessels have a power of "increase within themselves, both in diameter and in length, "which is according to the necessity, whether natural or dis-"eased."

"I shall now make some further observations relative to the external external and internal coagula of blood, and the intermediate one of lymph, which, if introduced before, would have too much interrupted the detail of the process. And first, of the external coagulum—

" Its particular figure and extent vary according to the manner in which the wound has been inflicted. If the artery, its sheath, the vein, and nerve accompanying it, have all been completely divided, the figure and extent of the coagulum will depend on the relative retraction of the artery to that of the sheath, which varies in different animals, and according as the artery has been more or less detached from the surrounding cellular substance.

" If the *artery alone* be divided, the anterior part of its sheath having been opened longitudinally, the coagulum which then forms at the mouth of the artery varies from a quarter to near half an inch in length, and differs from the form of the artery only in being sometimes slightly conical at its extremity; which, when the integuments have been sewed up previous to the division of the vessel, is turned forward, and is continuous with a large globular portion of coagulum, which being confined by the integuments alone, lies over it and the extremity of the artery. This appearance of the external coagulum may readily be accounted for from the circumstance of the sheath not having been divided so as to admit of its retraction; whereas, the complete division of the artery allows it to retract even within the part to which the wound of the sheath has extended, and, accordingly, that portion of the coagulum which lies within the entire part of the sheath, assumes the form of the artery; while the portion of sheath which has been opened, being deprived of the support of the artery, and of its tension, is more or less disposed to collapse, and will necessarily give a conical appearance to the portion of coagulum formed within it; and thus it is that the lower extremity of the external coagulum is sometimes conical: but when the integuments have been sewed up previous to the division of the artery, in consequence of the impediment which that circumstance affords to the exit of the blood, even this flaccid portion of the sheath becomes fully distended with blood, and of course the conical form of the external coagulum is prevented.—I have been thus particular in explaining these circumstances, because I wish it to be clearly understood, how a slight difference in the manner of performing the experiments may occasion a variation in the appearance of the parts when examined; and if, in repeating experiments on this subject, any deviation from the account here given should be observed, I am convinced it will be satisfactorily accounted for, by attending to the manner in which the experiment is performed in both instances.

" The extent, and, indeed, even the formation of the *internal* coagulum of blood, depends very much on the distance from a lateral branch, at which the division of the artery has taken place: thus, if it is divided about a quarter of an inch beyond a branch, there will scarcely be formed any coagulum of this description, for the effusion of lymph at the cut extremity of the artery is in general sufficient to form a coagulum which extends a little way within the artery, and then the space between the extremity of the coagulum of lymph and the lateral branch is so short, that no internal coagulum of blood can be formed, at least none worth mentioning. In some instances I have found a small lamina of coagulated blood, not thicker than a six-pence, lying on the coagulum of lymph, the extremity of which in these cases generally projects a little beyond the extremity of the artery, and extends further within its canal than merely the surface by which it adheres; which circumstance seems to depend on a larger quantity of lymph being effused than is necessary to fill up the canal of the artery as far as the inflammation extends on its internal surface, and the superfluous quantity not coming in contact with an inflamed surface, and the blood being constantly driven between it and the sides of the artery, it forms no adhesion, but projects a little within the canal. It is probable that the compression, which the lymph undergoes from the gradual contraction of the extremity of the artery, may also contribute to this effect.

"But when the division of an artery has taken place at some distance from a lateral branch, a long conical internal coagulum is then formed, whose base is situated towards the extremity of the artery, and in general it adheres partially at the circumference of its base to the internal surface of the artery, close to the coagulum of the lymph.

"The internal coagulum of blood, however, does not fill up the cavity of the artery throughout the whole of its extent; and though conical, it has often the appearance of not having been formed at once, but by the successive coagulation of small quantities of blood. It is very readily distinguished from the coagulum formed by the effusion of lymph from the inflamed extremity of the artery, which is rather brown than white at first, probably in consequence of some admixture of red particles, and which is *principally characterized by adhering almost throughout its whole extent to the internal surface of the artery:* whereas the former, *i. e.* the internal coagulum of blood, although much longer than this of lymph, forms no adhesion whatever, except a slight one at its base, and which seems to be produced by the coagulum of blood being formed while the lymph is sufficiently recent to allow it to stick to it. Although the internal coagulum of blood when first formed by no means fills up the canal of the artery, except at its base; yet, in consequence of the contraction which the portion of artery containing it gradually undergoes, after a short time it embraces the coagulum so closely that they appear to cohere to each other; so that although the greater part of the coagulum, preserving its natural form, may very easily be be separated from the artery, yet its internal surface is left of a black colour, as if an external lamina of the coagulum still remained on it. It is also highly worthy of notice, that on examining, at a distant period after these experiments, arteries which, (from similar experiments on the corresponding arteries of other animals more speedily examined), we know must have had considerable coagula of blood in them; no coagula should be found in them; but their internal surface is very black, and their external appearance, previous to being cut open, remarkably dark.

"From what has been said it appears, that when an artery has been divided at some distance from a lateral branch, three coagula are formed: one of blood, externally, which shuts up its mouth; one of lymph, just within the extremity of its canal; and one of blood, within its cavity, and contiguous to that of lymph.

"I have called that of lymph a coagulum, becaufe, when the divided artery has been left entirely to itfelf, there is fuch a quantity of lymph effufed, that although it is firmly united to the internal furface of the artery, it may be confidered as a diftinct fubstance; but, if the cut edges of the extremity of the artery had been kept in contact with each other by pressure, they would have cicatrized, and no coagulum would have been formed; *i. e.* coagulating lymph would not have been effused in such a quantity as to form a mass of a determinate figure.

"I have already remarked, that when the division of an artery has been made very near to a lateral branch, no internal coagulum of blood is formed: hence we see that the number of coagula varies according to circumstances. But the external coagulum is always formed, and is subject to no other variations than those already defcribed.

"The internal coagulum of blood contributes nothing to the suppression of hemorrhage in ordinary accidents, because its formation is uncertain, or when formed it rarely fills the canal of the artery, or, if it fills the canal, does not adhere to the internal coat of the artery. Hitherto, therefore, I have contented myfelf with noticing its existence, or pointing out the circumstance which prevents its formation, without ranking it amongst the means which Nature employs for the suppression of hemorrhage. But if an artery be lacerated, its internal coat will be torn in many places, in proportion to the degree of violence with which the injury has been inflicted. Under this particular accident the internal coagulum of blood may extend beyond many collateral branches, will fill the canal of the artery, and will adhere to its surface wherever it is lacerated, in consequence of lymph being effused from these several wounds of the internal coat. The internal coagulum may in this case avail against a return of hemorrhage."

The experiments, without doubt, bear our author through all the proceffes he has remarked, and even thro' the caufes to which he affigns thofe proceffes. But it is not enough to fhow, that coagulum on the part external to the orifice of the divided artery is the immediate and temporary means of ſuppreſſing the hemorrhage, or that the coagulum within the orifice is, for the moſt, accidental or unneceſſary, or even that the future and permanent ſuppreſſion of the hemorrhage ariſes from the effuſion of coagulable lymph at the divided extremities of the internal coat of the veſſels. If our Readers (and we are ſure all who are worth the labour we have willingly beſtowed on this article will) take the trouble of re-peruſing our extracts, as far as the quotation from Mr. Hunter, they will enjoy a ſecond time the deſcription given by our author of the changes produced in conſequence of ſo ſerious a violence as the diviſion of a conſiderable artery; and they will alſo feel, as the author ſeems to us to have done, not perfectly ſatisfied with the cauſes he aſſigns for ſome of the operations he obſerved.

The firſt effect of ſuch an injury is the coagulation of the blood to ſtop the orifice. This the author ſeems to impute (we admit with ſome caution) to the diminiſhed force of the circulation. We ſhould rather impute it to the reſources of the economy to preſerve itſelf under certain circumſtances. We alſo admit, that theſe reſources are not always ſufficient; but ſuch is the caſe in many other curative intentions of Nature. We well know that the bare ſtagnation of blood, either within or without the veſſels, provided it is ſtill encloſed within living animal matter, is not ſufficient to produce coagulation. This is often proved by large portions of blood in a grumous ſtate, as it is called, which ſometimes forms the ſubſtance of tumours ariſing probably from ruptured veins. Mr. Hunter gives ſeveral other inſtances, in which blood, both extravaſated in the living body, and remaining ſtagnated in its proper veſſels, did not coagulate. He alſo ſhows other inſtances, in which coagulation took place much higher up in the arteries, than the ſpace between the obſtructed part and any collateral branch. In this latter caſe, mortification had commenced at the lower extremity, and was ſpreading upward. A *neceſſary* conſequence of mortification would be a ſeparation of the mortified part and a conſequent hemorrhage. To prevent this, the blood coagulates in the veſſels. When an artery is punctured, ſo as to form what is called the ſpurious aneuriſm, or when the coats of an artery give way in the true aneuriſm, coagulum is formed over the injured or divided part, and, in ſome inſtances, has been found ſufficient to remove the diſeaſe by the obliteration even of a conſiderable veſſel.

As, therefore, in ſo many inſtances, coagulation does not follow extravaſation where it would be injurious to the animal, and does take place in ſo many others where it is abſolutely neceſſary for the preſervation of the animal's life; are we not authorized to ſay that it depends on the neceſſity the parts are under for the ſupport of life, and ſeems regulated by it? Are we not authorized in adopting Mr. Hunter's language, and conſidering coagulation in theſe inſtances, as *an action of the blood induced by the ſtimulus of neceſſity*

*ceſſity* in the ſurrounding parts? In one of our author's experiments, he found that a tea cup full of blood taken immediately after the inciſion into the carotid of a horſe, coagulated in five minutes and a few ſeconds; the ſame quantity, taken a quarter of an hour after, when the *neceſſity* became more urgent, as the animal was much enfeebled, coagulated in three minutes and a half. This ſpeedier coagulation could not ariſe from any diſpoſition to inflammation from greater powers in the animal; but from an ineffectual attempt at ſuppreſſing the hemorrhage; an attempt, however, which might have been ſuccefsful, had all the other reſources been allowed their full effect. We are told, the ſtream of blood had diminiſhed; the horſe fell; ſhowed great ſigns of reſtleſsneſs; and, to prevent miſchief, was diſpatched when in articulo mortis.

The quotation from Mr. Hunter is beautifully illuſtrated by the picture contained in our author's deſcription. We mean not to ſpeak of this enlargement of the collateral branches of an obliterated veſſel as a diſcovery of late times; it muſt have been known as long as the deſtruction of an artery was known. But we wiſh our readers to remark, that this altered action can only be imputed to the ſame ſtimulus of neceſſity, and is in perfect harmony with every other proceſs we perceive in the blood and the veſſels; that is, the parts act according to a neceſſity which ariſes for preſerving the whole animal, ſo that we may, without any ſtrain of words, be allowed to impute ſuch actions to the laws conſtituted for this particular ſtate of the animal.

The copious extracts we have already made, and the cloſe reaſoning required to accompany us through theſe obſervations, induce us, at preſent, reluctantly to attend to the limits of our work, and to the patience of our readers. In the next number the ſubject will be concluded.

---

*An Eſſay on the Effects of Carbonate of Iron upon Cancer, with an Inquiry into the Nature of that Diſeaſe.* By Richard Carmichael, Member of the Royal College of Surgeons, and Surgeon to St. George's Hoſpital and Diſpenſary. 8vo. 106 pages. 1806.

This work begins with a complaint on the *ennui*, occaſioned by the peruſal of modern medical productions. For our own parts, we find, with a very few exceptions, ſtill greater *ennui* in the peruſal of the ancients. To relieve his reader, however, Mr. Carmichael begins, by relating his caſes: theſe are certainly very ſtriking, and ſo well worth recording, that we ſometimes wiſh the author had reſerved his ſubſequent reaſoning till he had more completely matured his theory. The caſes are five in number. The firſt is of a woman who had a foul ulcer on each ſide of her noſe, ſimilar to the deſcription uſually given of *noli me tangere*; theſe were healed by the internal uſe of cicuta and calomel and the ſprinkling of hydrargyrus muriatus ruber on the parts; but in about two months afterwards, a foul ulcer appeared on the lip, which could not be relieved by the fame remedies. This induced the author to confult the modern writers on the fubject. By fome accident he was directed to Dr. Adams's work, which our readers will recollect is confined to the cancerous breaft.

"The grounds on which Doctor Adams founded his arguments in favour of the independent life of Cancer, feemed to me not unreafonable, although between that difeafe and Hydatids, I could not perceive any very great fimilarity. But this obvioufly led me to the confideration, that if the lives of thofe fuppofed animals were extinguifhed, they would be expelled from the body by fuppuration, — and as iron has been found to be very effectual in deftroying inteftinal worms, I was induced to hope, that it would be equally deftructive to other animals of a parafitical nature.—I, therefore, felt myfelf juftified in making trial of a madicine in itfelf harmlefs, the effects of which more than anfwered my expectations."

By the hiftory of the cafe, it appears not only that the carbonate of iron was fuccefsful, but alfo that as often as that remedy was omitted, by any accident, before the compleat cicatrization of the fore, the ulcer conftantly fpread, and became ill-conditioned. During the cure, we are told, the ulcer occafionally difcharged fubftances about the fize of the fmalleft pea.

The fecond cafe was a cancerous ulcer in the external canthus of the eye; this was healed by the fame remedies, carbonate of iron: and in the progrefs, the author remarked "fmall cavities refembling thofe greater ones obfervable in iffues after the removal of the pea." This appearance, he concludes, was owing to the difcharge of fmall bodies, fimilar to thofe mentioned in the former cafe.

The third cafe was an ulcer of that *fpecies*, called *Noli me tangere*, in a very young lady. Before we proceed any further, we cannot help confeffing, that we have never met with this difeafe in a very young fubject. However, the carbonate of iron cured the complaint in fix days, which had withftood, for as many years, the moft *powerful* remedies. This ulcer does not appear to have difcharged any of thofe round fubftances.

The fourth cafe was of a man, forty years of age, with cancerous ulcers on the fcrotum and on the calf of the leg, the latter covered with a number of warty excrefcences. Thefe were all difcharged by the carbonate of iron, and both the ulcers yielded to the ufe of that remedy applied in various ways.

The fifth cafe was of a foldier nearly eighty years of age.—Here alfo the remedy was fuccefsful; but it will eafily be conceived the patient did not long outlive the cure of his local complaints.

After the relation of his Cafes, Mr. Carmichael introduces his reafoning by an account of what has been done by the ancients. This begins with Hippocrates, and is purfued as far down as Helvetius. Whether all the intermediate writers are included, we did not think it neceffary to enquire, confidering how little was to be learned from thofe who are quoted.

In

In reviewing the moderns, we are informed of Boerhaave's infpiffating, Heifter's coagulating theory, Le Dran's and Pearfon's obftructions. We think our author too fevere on Darwin, whofe hiftory of the formation of Scirrhus and Cancer is certainly not bad for a poet.

" But," fays our author, " an hypothefis has been lately revived by Dr. Adams, which affigns to cancer a life independent of the part in which it is fituated." It is well known that Dr. A. affigns to carcinoma that fpecies of life which has been detected in hydatids. We, therefore, expected to have learned who are the writers from whom Dr. A. received thofe opinions, which he is faid to have revived. Inftead of this, we are introduced to the fmall worms mentioned in the Bibliotheca Anatomica; the infects which Juftamond conceives, might be admitted at the external pores of the breaft, and which fome of the French furgeons *had feen*. All this is furely very different from the nature of hydatids.

Many other remarks follow, which evidently prove that Mr. Carmichael has only an imperfect knowledge of Dr. Adams's Theory; however, in the midft of all this, we meet with one important objection. " Thefe appearances," fays the author, " feem to be merely the effects of the deranged animal economy, excepting the one evincing a contractile power, in what he (Dr. A.) calls the capfules, but which, notwithftanding repeated inveftigations, I never could perceive. The colour of the fat, which Dr. A. feems to lay fo much ftrefs upon, may probably be produced by animal hepatic air, which Dr. Crawford has proved to be " capable of imparting to the fat of recently killed animals a green colour;" and that this very air, united to ammonia, efcapes in great abundance from the cancerous as well as other malignant ulcers." The colour of the fat we do not recollect that Dr. A. lays fo much ftrefs on; becaufe he remarks, that when cold, it has all the properties of common fat. But if Mr. Carmichael has carefully inveftigated that fubftance when recently taken from a cancerous breaft, and found none of the papillary appearance mentioned by Dr. A. he is certainly authorized in rejecting the theory.

" As to the cells in which this fubftance is depofited, and which feem fo ftrong, that without breaking them, their contents may be dug out; they appeared to me on diffection, to be common cellular membrane, partially fupported by the ligamentous bands, or what he terms the fungus, but which had lefs the appearance of loculamenta containing a liquid, than the roots of a plant penetrating where there was leaft refiftance — but with every attention, I could not difcover the papillary appearance from which Doctor Adams deduces the proofs of the contraction and the evidence of the life Cancer.—But if any motion fhould be obferved by future inveftigators, the vitality may be attributed with as much reafon to the cartilaginous as to the pituitous fubftance."

Here alfo, if Mr. Carmichael, in attempting to remove the fat from thefe cells, found it fo univerfally enveloped with the common cellular fubftance, that he could not feparate it without breaking the cells as in common fat, he is fairly at iſſue with Dr. A. and the controverſy muſt remain open for future enquiry.

The fifth chapter contains "Enquiries into the Nature of Cancer." Here our author again begins with Dr. Adams, whoſe opinions, he obſerves, are, notwithſtanding the objections he has made, conſonant with his own, as to the independent life of cancer, though he differs as to the ſeat of that life. We moſt ſincerely wiſh he had followed a plan, firſt, we believe, introduced by Dr. A. and ſince adopted by moſt ſucceeding writers on this ſubject, that is, not only to tell us what he means by cancer, but alſo to make ſome diviſion, according to the different ſtructures of diſeaſed parts, generally conſidered cancerous. It is remarkable, that none of the caſes he gives ſeem to have the ſmalleſt analogy with that which is the ſubject of Dr. Adams's treatiſe; yet in his enquiry he ſeems to include every thing that has ever been denominated cancer; and in ſo doing, has no difficulty in proving the ſeparate vitality of cancer.

"The origin of carcinoma, ſays Mr. C. firſt commencing in a point; the formation of cyſts in its texture; theſe cyſts evincing a contractile power by a forceable expulſion of their contents on being punctured; and the peculiar pain in this diſeaſe, are all circumſtances which ſtrongly impreſs the idea that carcinoma is poſſeſſed of individual life.

"The force with which the fluid is expelled from the cyſts, is undoubtedly far greater than the ſimple elaſticity of the part is capable of effecting and it is not a little ſurpriſing that ſome deduction has not been already drawn from this circumſtance: but as thoſe who have not witneſſed the fact, will be pleaſed with a corroboration of my aſſertion, ſhall recite a paſſage from Le Dran, who, endeavoring to demonſtrate the infectious nature of cancer, accidently mentions a ſtrong proof the contractile power of thoſe cyſts. He relates, "that in the middle of a carcinomatous tumour "extirpated by his father, there was a cyſt filled with a fluid, "which he opened, and part of its contents *ſpurting out* upon his "clothes, deſtroyed the colour of them, as if it had been aqua-"fortis; ſome of it *flew in his face*, and he felt continued ſhootings "there for ſeveral hours, though he immediately waſhed the part." Therefore the ſtrong force with which theſe cyſts expel their contents, is not owing to their exceſſive repletion, but entirely to the action of fibres endued with a contractile power. Such a power, even in a ſmaller, degree is eſteemed ſufficient to eſtabliſh the animality of hydatids generated in the human body, and on the ſame grounds the vitality of Cancer may likewiſe be inferred."

It would have been ſurpriſing indeed, if Dr. Adams had overlooked this circumſtance in ſupporting his favourite hypotheſis. The mention of muſcular power in cyſts, containing fluids of various deſcriptions, and their frequent occurrence in parts which are found the moſt favourable nidus for what he calls carcinomatous hydatids, occurs in ſeveral parts of Dr. A's. treatiſe. However, we truſt that gentleman will feel the obligations he owes Mr. Carmichael for this quotation from Le Dran.

The

The fucceeding chapter is on the treatment of cancer, in the courfe of which the author feems willing to believe that iron is injurious to all animals whofe blood is without red particles. When we confider how univerfally this mineral is diffufed throughout nature, we cannot but hefitate before we admit of fuch a conclufion. If it really is the cafe that iron deftroys fuch animals as are without red blood, we fhall be in poffeffion of a remedy more valuable than we need expatiate on. Independent of all our doubts concerning the carcinomatous difpofition in the cafes defcribed by our author, the remedy appears to be a very valuable one, and we truft, by degrees, its full merits will be appreciated.

A Poftfcript follows, in which more cafes are given on the authority of Dr. C. and his brother practitioners; though they are ot fufficiently numerous to eftablifh the credit of his remedy in fo ftubborn a difeafe, we cannot conclude without acknowledging our obligation to the author for his communication.

---

*A Practical Treatife on various Difeafes of the Abdominal Vifcera*. By C. R. Pemberton, M. D. F. R. S. Fellow of the College of Phyficians, &c. &c. 8vo. pp. 180. London, 1806.

Works on fubjects of medicine or furgery, which embrace a confiderable fcope, and treat of a variety of difeafes, commonly contain much that may be found in older authors. This, perhaps, is fcarcely to be avoided, when the writer's object is to give a full enumeration of all the fymptoms, and all the remedies that have been found efficacious in each difeafe. Dr. P. in the truly original and fcientific work before us, has no fuch object; and it is his profeffed defign, to copy nothing from his predeceffors.

"I have endeavoured, (fays he) in the following work, to trace thefe fymptoms with fome minuteness and precision, and I truft that the younger Profeffors of our art, will here find materials of practical knowledge, by which they will be enabled to form a juft and early prognoftic of the nature and tendency of internal diforders.

"The Obfervations, which are here fubmitted to the attention of the public, are the refult of feveral years experience: they were made at the bed-fide of the Patient, and were faithfully recorded at the time, when the cafes were paffing under my attention.

"As I do not mean to introduce any matter, which has not been the refult of my own practice, or which has not arifen from my own experience in the practice of others, the reader muft not expect to find in this work a regular hiftory of the abdominal difeafes, as they are recorded by our authors, who have collected the opinions of others on this ample and important theme.

"I muft obferve, however, that as I have meditated on this fubject for a confiderable period, and loft no opportunity of obfervation during life, or refearch after death, I am difpofed to imagine that this little volume will be found to contain fome remarks, not altogether unworthy of attention, even to the experienced Practitioner, upon almoft every diforder of the Abdominal Vifcera: and he he will, I truft, not fail to difcover a vein of enquiry into certain difeafes, which others have but flightly recorded, or inadequately conceived."

The following organs and parts of the abdomen, with their acute and chronic difeafes, are treated of in this work, viz. The peritonæum, the liver, the gall bladder, the pancreas, the fpleen, the kidneys, the ftomach, and the inteftines.

In treating of inflammation of the liver, our author is naturally led to the fubject of venæfection, and after feveral important obfervations on the appearance of the blood drawn, he adds:

"Phyficians have been ftruck, at all times, with the effect produced by taking the blood from a large orifice in inflammatory difeafes, and it is certainly a matter which cannot be too ftrongly urged as an indifpenfable point in practice . efpecially as the generality of writers do not feem to have inftituted any defined plan to regulate and fecure the effectual performance of this important operation. I wifh, therefore, to prefs in the ftrongeft terms the abfolute neceffity of attending to that circumftance, which the following obfervations may perhaps tend to elucidate.

"It is true, that from a *fmall* orifice an equal quantity of blood may be taken as from a *large* one, but the *time* of its flowing is fo long, that the topical inflammation, which demands for its relief a fudden effect upon the fyftem, is not in the leaft influenced by it, though the general ftrength is much weakened, which is an occurrence of all others to be avoided in a difeafe, that requires repeated evacuations.

"As I confider this matter of great confequence, I fhall endeavour to point out a method, by which a plan of a more defined nature than that hitherto adopted may be given for drawing blood in inflammatory difeafes.

"At prefent we are contented to order, that the blood fhould be taken from a large or from a fmall orifice, than which nothing furely can be more vague or undefined. The plan, which I propofe, refers to the *length of time* in taking the blood, which may be meafured, and not to the fize of the orifice, which can not.

"I find from numerous experiments, made at my defire for this purpofe by different furgeons, that when the orifice is fuch as to permit eight ounces of blood to flow in three minutes, that then, a patient under acute inflammation will receive every benefit which is expected from the remedy. If it flows in a longer time he will receive lefs benefit, and under certain circumftances no benefit at all, or even an abfolute injury.

"I can fuppofe a cafe of peripneumony, where a patient fhall have juft general ftrength enough to carry on refpiration by the affiftance of the voluntary mufcles, and that eight ounces of blood fhall be taken from a very fmall orifice, by which the change will be fo gradual, in confequence of the time required for the blood to flow, that no alteration whatever will be made in the difeafe, but yet the general ftrength fhall be fo diminifhed, that death will enfue. On the other hand, had the fame quantity of blood been taken taken by a large orifice, that *then* the diſeaſe would have felt the remedy, and reſpiration would have gone on with leſs exertion of the remaining general ſtrength, in conſequence of the lungs being relieved by this ſudden depletion.

"The great conſequence, therefore, attached to the mode in which blood is drawn, (as on this alone life or death may often depend) imperiouſly demands of every phyſician to impreſs upon the mind of his patient the importance of the operation, and the abſolute neceſſity of having it performed by a perſon fully ſkilled in his profeſſion.

"I ſhould not omit to mention, that there may now and then occur a caſe, where the vein may not only be particularly ſmall, but likewiſe be deep ſeated, and covered with fat. Here, altho' the orifice may be ſufficiently large, yet a portion of fat will obtrude, ſo as to interrupt the ſtream of blood. I would in ſuch caſe recommend the ſurgeon to dilate the external orifice, or even make a freſh orifice, rather than perſiſt in his endeavours to obtain the quantity required in this gradual way."

We have given theſe obſervations in the Author's own words, without abridgment, becauſe they appear to us of conſiderable importance in practice.

( To be concluded in our next. )

